# (*E*)-3-(2-Chloro­phen­yl)-1-(3-methoxy­phen­yl)prop-2-en-1-one

**DOI:** 10.1107/S1600536808021910

**Published:** 2008-07-19

**Authors:** Hoong-Kun Fun, Samuel Robinson Jebas, P. S. Patil, S. M. Dharmaprakash

**Affiliations:** a X-ray Crystallography Unit, School of Physics, Universiti Sains Malaysia, 11800 USM, Penang, Malaysia; b Department of Studies in Physics, Mangalore University, Mangalagangotri, Mangalore 574 199, India

## Abstract

The title compound, C_16_H_13_ClO_2_, adopts an *E* configuration with respect to the double bond of the propenone unit. The two benzene rings are twisted slightly from each other, making a dihedral angle of 7.14 (5)°. The mol­ecules are arranged in stacks, in which adjacent mol­ecules are related by inversion symmetry and form π–π inter­actions with a centroid–centroid distance of 3.7098 (6) Å. C—H⋯O and C—H⋯π inter­actions are formed between neighbouring mol­ecules.

## Related literature

For related literature, see: Chantrapromma *et al.* (2005 ▶, 2006 ▶); Fun *et al.* (2006 ▶); Patil, Fun *et al.* (2007 ▶); Patil, Dharmaprakash *et al.* (2006 ▶, 2007 ▶).
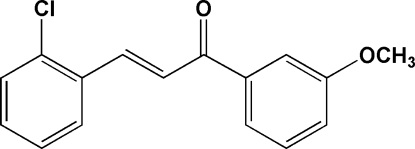


## Experimental

### 

#### Crystal data

C_16_H_13_ClO_2_
*M* *_r_* = 272.71
Triclinic, 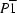

*a* = 7.7352 (2) Å
*b* = 8.1405 (2) Å
*c* = 10.7411 (2) Å
α = 87.392 (1)°
β = 82.147 (1)°
γ = 74.794 (1)°
*V* = 646.52 (3) Å^3^
*Z* = 2
Mo *K*α radiation
μ = 0.29 mm^−1^
*T* = 100.0 (1) K
0.38 × 0.30 × 0.16 mm

#### Data collection

Bruker SMART APEXII CCD diffractometer
Absorption correction: multi-scan (*SADABS*; Bruker, 2005 ▶) *T*_min_ = 0.899, *T*_max_ = 0.955
17497 measured reflections
4314 independent reflections
3644 reflections with *I* > 2σ(*I*)
*R*_int_ = 0.025

#### Refinement

*R*[*F* ^2^ > 2σ(*F* ^2^)] = 0.037
*wR*(*F* ^2^) = 0.101
*S* = 1.06
4314 reflections
173 parameters
H-atom parameters constrained
Δρ_max_ = 0.53 e Å^−3^
Δρ_min_ = −0.23 e Å^−3^

### 

Data collection: *APEX2* (Bruker, 2005 ▶); cell refinement: *SAINT* (Bruker, 2005 ▶); data reduction: *SAINT*; program(s) used to solve structure: *SHELXTL* (Sheldrick, 2008 ▶); program(s) used to refine structure: *SHELXTL*; molecular graphics: *SHELXTL*; software used to prepare material for publication: *SHELXTL* and *PLATON* (Spek, 2003 ▶).

## Supplementary Material

Crystal structure: contains datablocks global, I. DOI: 10.1107/S1600536808021910/bi2296sup1.cif

Structure factors: contains datablocks I. DOI: 10.1107/S1600536808021910/bi2296Isup2.hkl

Additional supplementary materials:  crystallographic information; 3D view; checkCIF report

## Figures and Tables

**Table 1:** Hydrogen-bond geometry (Å, °) *Cg*1 is the centroid of the C1–C6 ring.

*D*—H⋯*A*	*D*—H	H⋯*A*	*D*⋯*A*	*D*—H⋯*A*
C2—H2*A*⋯O2^i^	0.93	2.50	3.3887 (13)	161
C4—H4*A*⋯O1^ii^	0.93	2.57	3.3003 (13)	136
C16—H16*B*⋯O1^iii^	0.96	2.58	3.4899 (14)	158
C16—H16*C*⋯*Cg*1^iv^	0.96	2.82	3.6137 (14)	135

Symmetry codes: (i) 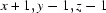; (ii) 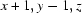; (iii) 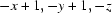; (iv) 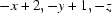.

**Fig. 1.**
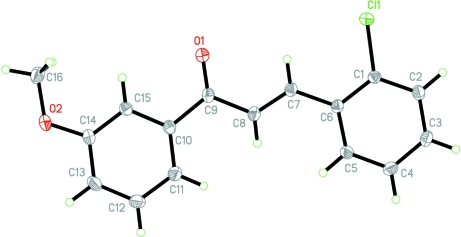
The molecular structure of the title compound, showing 50% probability displacement ellipsoids for non-H atoms.

**Fig. 2.**
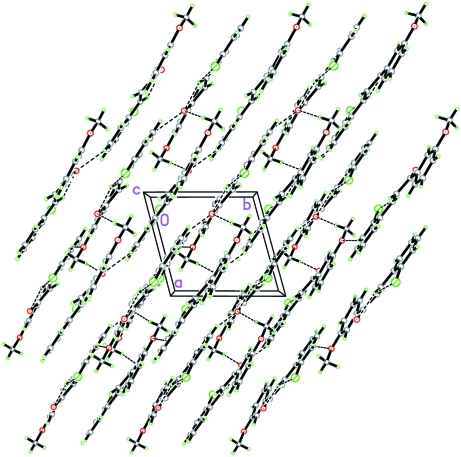
Crystal packing of the title compound, viewed along the c axis. Hydrogen bonds are shown as dashed lines.

## supplementary crystallographic information

### Comment

As a part of our ongoing investigation of non-linear optical (NLO) compounds (Chantrapromma *et al.*, 2005, 2006; Fun *et al.*, 2006; Patil, Fun *et al.*, 2007; Patil, Dharmaprakash *et al.*, 2006, 2007), the title compound has recently been prepared in our laboratory and its crystal structure is presented here.

The molecule exhibits an *E* configuration with respect to the C7=C8 double bond, the torsion angle being C7—C8—C9—C10 = -166.3 (9)°. The dihedral angle between the two phenyl rings is 7.14 (5)°, indicating that they are slightly twisted from each other. The molecules are interconnected by weak C—H···O interactions and the packing is further consolidated by C–H···π and π–π interactions between the C1—C6 (centroid *Cg*1) and C10—C15 (centroid *Cg*2) rings: *Cg*1···*Cg*2^i^ = 3.7098 (6) Å [symmetry code: (i) 2 - *x*, -*y*, -*z*].

### Experimental

2-Chlorobenzaldehyde (0.01 mol, 1.13 g) in ethanol (20 ml) was mixed with 3-methoxyacetophenone (0.01 mol, 1.37 ml) in 20 ml ethanol and the mixture was treated with 10 ml of 10% sodium hydroxide solution and stirred at room temperature for 8 h. The precipitate obtained was poured into ice-cold water (500 ml) and left to stand for 5 h. The resulting crude solid was filtered, dried and recrystallized from *N*,*N*-dimethylformamide by slow evaporation.

### Refinement

H atoms were positioned geometrically with C—H = 0.93Å or C_methyl_—H = 0.96 Å and refined using a riding model, with *U*_iso_(H) = 1.2*U*_eq_(C) or 1.5_eq_(C_methyl_). A rotating group model was used for the methyl groups.

### Crystal data

**Table:**

C_16_H_13_ClO_2_	*Z* = 2
*M**_r_* = 272.71	*F*_000_ = 284
Triclinic, *P*1	*D*_x_ = 1.401 Mg m^−^^3^
Hall symbol: -P 1	Mo *K*α radiation λ = 0.71073 Å
*a* = 7.7352 (2) Å	Cell parameters from 7792 reflections
*b* = 8.1405 (2) Å	θ = 2.2–37.5º
*c* = 10.7411 (2) Å	µ = 0.29 mm^−^^1^
α = 87.392 (1)º	*T* = 100.0 (1) K
β = 82.147 (1)º	Block, colourless
γ = 74.794 (1)º	0.38 × 0.30 × 0.16 mm
*V* = 646.52 (3) Å^3^	

### Data collection

**Table:**

Bruker SMART APEXII CCD diffractometer	4314 independent reflections
Radiation source: fine-focus sealed tube	3644 reflections with *I* > 2σ(*I*)
Monochromator: graphite	*R*_int_ = 0.025
*T* = 100(1) K	θ_max_ = 31.7º
φ and ω scans	θ_min_ = 2.6º
Absorption correction: multi-scan(SADABS; Bruker, 2005)	*h* = −11→10
*T*_min_ = 0.899, *T*_max_ = 0.955	*k* = −11→12
17497 measured reflections	*l* = −15→15

### Refinement

**Table:**

Refinement on *F*^2^	Secondary atom site location: difference Fourier map
Least-squares matrix: full	Hydrogen site location: inferred from neighbouring sites
*R*[*F*^2^ > 2σ(*F*^2^)] = 0.037	H-atom parameters constrained
*wR*(*F*^2^) = 0.101	*w* = 1/[σ^2^(*F*_o_^2^) + (0.0513*P*)^2^ + 0.1689*P*] where *P* = (*F*_o_^2^ + 2*F*_c_^2^)/3
*S* = 1.06	(Δ/σ)_max_ = 0.001
4314 reflections	Δρ_max_ = 0.53 e Å^−^^3^
173 parameters	Δρ_min_ = −0.23 e Å^−^^3^
Primary atom site location: structure-invariant direct methods	Extinction correction: none

### Special details

**Table:**

Geometry. All e.s.d.'s (except the e.s.d. in the dihedral angle between two l.s. planes) are estimated using the full covariance matrix. The cell e.s.d.'s are taken into account individually in the estimation of e.s.d.'s in distances, angles and torsion angles; correlations between e.s.d.'s in cell parameters are only used when they are defined by crystal symmetry. An approximate (isotropic) treatment of cell e.s.d.'s is used for estimating e.s.d.'s involving l.s. planes.
Refinement. Refinement of *F*^2^ against ALL reflections. The weighted *R*-factor *wR* and goodness of fit *S* are based on *F*^2^, conventional *R*-factors *R* are based on *F*, with *F* set to zero for negative *F*^2^. The threshold expression of *F*^2^ > σ(*F*^2^) is used only for calculating *R*-factors(gt) *etc*. and is not relevant to the choice of reflections for refinement. *R*-factors based on *F*^2^ are statistically about twice as large as those based on *F*, and *R*- factors based on ALL data will be even larger.

### Fractional atomic coordinates and isotropic or equivalent isotropic displacement parameters (Å^2^)

**Table:**

	*x*	*y*	*z*	*U*_iso_*/*U*_eq_	
Cl1	1.15204 (4)	0.08594 (3)	−0.41471 (2)	0.02216 (8)	
O1	0.78728 (11)	0.44819 (10)	−0.05788 (8)	0.02200 (17)	
O2	0.46589 (11)	0.70028 (11)	0.36344 (8)	0.02281 (17)	
C1	1.29054 (14)	−0.01282 (13)	−0.30363 (10)	0.01529 (19)	
C2	1.44112 (15)	−0.14294 (13)	−0.34590 (10)	0.0184 (2)	
H2A	1.4678	−0.1705	−0.4307	0.022*	
C3	1.55059 (15)	−0.23075 (13)	−0.25988 (11)	0.0196 (2)	
H3A	1.6517	−0.3178	−0.2870	0.024*	
C4	1.51011 (15)	−0.18945 (13)	−0.13323 (11)	0.0189 (2)	
H4A	1.5830	−0.2495	−0.0755	0.023*	
C5	1.36046 (14)	−0.05817 (13)	−0.09349 (10)	0.0172 (2)	
H5A	1.3348	−0.0311	−0.0086	0.021*	
C6	1.24650 (14)	0.03518 (12)	−0.17720 (10)	0.01485 (19)	
C7	1.09255 (14)	0.17825 (13)	−0.13473 (10)	0.01589 (19)	
H7A	1.0407	0.2526	−0.1957	0.019*	
C8	1.02124 (15)	0.20974 (13)	−0.01471 (10)	0.0173 (2)	
H8A	1.0667	0.1341	0.0477	0.021*	
C9	0.87148 (14)	0.36241 (13)	0.02113 (10)	0.01643 (19)	
C10	0.82784 (14)	0.40997 (13)	0.15679 (10)	0.01584 (19)	
C11	0.94385 (16)	0.34122 (14)	0.24498 (11)	0.0207 (2)	
H11A	1.0518	0.2604	0.2210	0.025*	
C12	0.89719 (18)	0.39431 (15)	0.36938 (11)	0.0248 (2)	
H12A	0.9748	0.3489	0.4284	0.030*	
C13	0.73691 (17)	0.51359 (14)	0.40576 (11)	0.0218 (2)	
H13A	0.7062	0.5474	0.4892	0.026*	
C14	0.62110 (15)	0.58335 (13)	0.31749 (10)	0.0173 (2)	
C15	0.66548 (14)	0.53249 (13)	0.19298 (10)	0.01602 (19)	
H15A	0.5882	0.5793	0.1341	0.019*	
C16	0.34703 (16)	0.78256 (16)	0.27587 (12)	0.0253 (2)	
H16A	0.2445	0.8619	0.3194	0.038*	
H16B	0.3070	0.6989	0.2355	0.038*	
H16C	0.4096	0.8423	0.2137	0.038*	

### Atomic displacement parameters (Å^2^)

**Table:**

	*U*^11^	*U*^22^	*U*^33^	*U*^12^	*U*^13^	*U*^23^
Cl1	0.02040 (14)	0.02714 (14)	0.01654 (13)	−0.00027 (10)	−0.00537 (9)	−0.00122 (9)
O1	0.0219 (4)	0.0231 (4)	0.0173 (4)	0.0008 (3)	−0.0021 (3)	−0.0019 (3)
O2	0.0212 (4)	0.0263 (4)	0.0173 (4)	−0.0009 (3)	0.0018 (3)	−0.0059 (3)
C1	0.0140 (4)	0.0170 (4)	0.0154 (4)	−0.0045 (4)	−0.0027 (4)	−0.0004 (3)
C2	0.0166 (5)	0.0204 (5)	0.0177 (5)	−0.0041 (4)	−0.0005 (4)	−0.0049 (4)
C3	0.0155 (5)	0.0170 (4)	0.0249 (5)	−0.0014 (4)	−0.0019 (4)	−0.0049 (4)
C4	0.0168 (5)	0.0179 (5)	0.0223 (5)	−0.0030 (4)	−0.0061 (4)	−0.0004 (4)
C5	0.0162 (5)	0.0197 (5)	0.0159 (5)	−0.0044 (4)	−0.0027 (4)	−0.0019 (4)
C6	0.0129 (4)	0.0159 (4)	0.0162 (5)	−0.0043 (3)	−0.0014 (3)	−0.0022 (3)
C7	0.0137 (4)	0.0161 (4)	0.0181 (5)	−0.0038 (3)	−0.0019 (4)	−0.0023 (3)
C8	0.0173 (5)	0.0150 (4)	0.0184 (5)	−0.0029 (4)	0.0000 (4)	−0.0006 (4)
C9	0.0152 (5)	0.0162 (4)	0.0176 (5)	−0.0046 (4)	0.0005 (4)	−0.0023 (3)
C10	0.0168 (5)	0.0158 (4)	0.0154 (4)	−0.0056 (4)	−0.0005 (4)	−0.0015 (3)
C11	0.0201 (5)	0.0191 (5)	0.0218 (5)	−0.0019 (4)	−0.0042 (4)	−0.0028 (4)
C12	0.0303 (6)	0.0228 (5)	0.0204 (5)	−0.0022 (5)	−0.0100 (5)	−0.0007 (4)
C13	0.0286 (6)	0.0216 (5)	0.0151 (5)	−0.0058 (4)	−0.0025 (4)	−0.0027 (4)
C14	0.0185 (5)	0.0166 (4)	0.0168 (5)	−0.0058 (4)	0.0013 (4)	−0.0028 (4)
C15	0.0163 (5)	0.0172 (4)	0.0150 (5)	−0.0056 (4)	−0.0009 (4)	−0.0008 (3)
C16	0.0173 (5)	0.0288 (6)	0.0272 (6)	−0.0010 (4)	−0.0022 (4)	−0.0070 (5)

### Geometric parameters (Å, °)

**Table:**

Cl1—C1	1.7400 (11)	C8—C9	1.4836 (15)
O1—C9	1.2208 (14)	C8—H8A	0.930
O2—C14	1.3684 (13)	C9—C10	1.4952 (14)
O2—C16	1.4264 (15)	C10—C11	1.3915 (15)
C1—C2	1.3917 (14)	C10—C15	1.4028 (14)
C1—C6	1.4018 (14)	C11—C12	1.3933 (16)
C2—C3	1.3849 (16)	C11—H11A	0.930
C2—H2A	0.930	C12—C13	1.3796 (17)
C3—C4	1.3899 (16)	C12—H12A	0.930
C3—H3A	0.930	C13—C14	1.3929 (16)
C4—C5	1.3863 (15)	C13—H13A	0.930
C4—H4A	0.930	C14—C15	1.3886 (14)
C5—C6	1.4021 (15)	C15—H15A	0.930
C5—H5A	0.930	C16—H16A	0.960
C6—C7	1.4680 (14)	C16—H16B	0.960
C7—C8	1.3391 (15)	C16—H16C	0.960
C7—H7A	0.930		
			
C14—O2—C16	117.57 (9)	O1—C9—C10	120.76 (10)
C2—C1—C6	122.55 (10)	C8—C9—C10	118.14 (9)
C2—C1—Cl1	116.93 (8)	C11—C10—C15	120.12 (10)
C6—C1—Cl1	120.49 (8)	C11—C10—C9	122.44 (10)
C3—C2—C1	119.02 (10)	C15—C10—C9	117.41 (9)
C3—C2—H2A	120.5	C10—C11—C12	119.56 (10)
C1—C2—H2A	120.5	C10—C11—H11A	120.2
C2—C3—C4	120.35 (10)	C12—C11—H11A	120.2
C2—C3—H3A	119.8	C13—C12—C11	120.58 (11)
C4—C3—H3A	119.8	C13—C12—H12A	119.7
C5—C4—C3	119.61 (10)	C11—C12—H12A	119.7
C5—C4—H4A	120.2	C12—C13—C14	119.96 (10)
C3—C4—H4A	120.2	C12—C13—H13A	120.0
C4—C5—C6	122.09 (10)	C14—C13—H13A	120.0
C4—C5—H5A	119.0	O2—C14—C15	124.45 (10)
C6—C5—H5A	119.0	O2—C14—C13	115.23 (10)
C1—C6—C5	116.37 (9)	C15—C14—C13	120.32 (10)
C1—C6—C7	122.05 (9)	C14—C15—C10	119.46 (10)
C5—C6—C7	121.56 (9)	C14—C15—H15A	120.3
C8—C7—C6	124.89 (10)	C10—C15—H15A	120.3
C8—C7—H7A	117.6	O2—C16—H16A	109.5
C6—C7—H7A	117.6	O2—C16—H16B	109.5
C7—C8—C9	121.58 (10)	H16A—C16—H16B	109.5
C7—C8—H8A	119.2	O2—C16—H16C	109.5
C9—C8—H8A	119.2	H16A—C16—H16C	109.5
O1—C9—C8	121.09 (10)	H16B—C16—H16C	109.5
			
C6—C1—C2—C3	1.08 (15)	O1—C9—C10—C11	−164.94 (10)
Cl1—C1—C2—C3	−176.96 (8)	C8—C9—C10—C11	14.25 (15)
C1—C2—C3—C4	0.12 (16)	O1—C9—C10—C15	13.15 (15)
C2—C3—C4—C5	−0.79 (16)	C8—C9—C10—C15	−167.66 (9)
C3—C4—C5—C6	0.30 (16)	C15—C10—C11—C12	0.30 (16)
C2—C1—C6—C5	−1.52 (15)	C9—C10—C11—C12	178.33 (10)
Cl1—C1—C6—C5	176.45 (7)	C10—C11—C12—C13	0.30 (18)
C2—C1—C6—C7	176.86 (9)	C11—C12—C13—C14	−0.69 (18)
Cl1—C1—C6—C7	−5.17 (13)	C16—O2—C14—C15	−3.86 (15)
C4—C5—C6—C1	0.82 (15)	C16—O2—C14—C13	176.68 (10)
C4—C5—C6—C7	−177.57 (10)	C12—C13—C14—O2	179.96 (10)
C1—C6—C7—C8	165.91 (10)	C12—C13—C14—C15	0.48 (17)
C5—C6—C7—C8	−15.79 (15)	O2—C14—C15—C10	−179.32 (9)
C6—C7—C8—C9	176.74 (9)	C13—C14—C15—C10	0.11 (15)
C7—C8—C9—O1	12.94 (16)	C11—C10—C15—C14	−0.50 (15)
C7—C8—C9—C10	−166.25 (9)	C9—C10—C15—C14	−178.64 (9)

### Hydrogen-bond geometry (Å, °)

**Table:**

*D*—H···*A*	*D*—H	H···*A*	*D*···*A*	*D*—H···*A*
C2—H2A···O2^i^	0.93	2.50	3.3887 (13)	161
C4—H4A···O1^ii^	0.93	2.57	3.3003 (13)	136
C16—H16B···O1^iii^	0.96	2.58	3.4899 (14)	158
C16—H16C···Cg1^iv^	0.96	2.82	3.6137 (14)	135

Symmetry codes: (i) *x*+1, *y*−1, *z*−1; (ii) *x*+1, *y*−1, *z*; (iii) −*x*+1, −*y*+1, −*z*; (iv) −*x*+2, −*y*+1, −*z*.
